# Role of Mitochondrial DNA Copy Number Alteration in Human Renal Cell Carcinoma †

**DOI:** 10.3390/ijms17060814

**Published:** 2016-05-25

**Authors:** Chen-Sung Lin, Hui-Ting Lee, Ming-Huei Lee, Siao-Cian Pan, Chen-Yeh Ke, Allen Wen-Hsiang Chiu, Yau-Huei Wei

**Affiliations:** 1 Faculty of Medicine, National Yang-Ming University, Taipei 112, Taiwan; doc2765c@ms59.hinet.net (C.-S.L.); htlee1228@gmail.com (H.-T.L.); 2 Institute of Clinical Medicine, National Yang-Ming University, Taipei 112, Taiwan; 3 Division of Thoracic Surgery, Taipei Hospital, Ministry of Health and Welfare, New Taipei City 242, Taiwan; 4 Division of Thoracic Surgery, Feng-Yuan Hospital, Ministry of Health and Welfare, Taichung City 420, Taiwan; 5 Department of Medicine, Mackay Medical College, New Taipei City 252, Taiwan; 6 Division of Allergy, Immunology and Rheumatology, Mackay Memorial Hospital, Taipei 104, Taiwan; k.elantris@gmail.com; 7 Division of Urology, Feng-Yuan Hospital, Ministry of Health and Welfare, Taichung City 420, Taiwan; 470916lee@gmail.com; 8 Institute of Biochemistry and Molecular Biology, National Yang-Ming University, Taipei 112, Taiwan; 9 Department of Life Sciences and Institute of Genome Sciences, National Yang-Ming University, Taipei 112, Taiwan; georgecyk@gmail.com; 10 Division of Urology, Taipei City Hospital, Ren-Ai Branch, Taipei 106, Taiwan

**Keywords:** renal cell carcinoma, mitochondrial DNA copy number, mitochondrial biogenesis, Warburg effect, invasiveness

## Abstract

We investigated the role of mitochondrial DNA (mtDNA) copy number alteration in human renal cell carcinoma (RCC). The mtDNA copy numbers of paired cancer and non-cancer parts from five resected RCC kidneys after radical nephrectomy were determined by quantitative polymerase chain reaction (Q-PCR). An RCC cell line, 786-O, was infected by lentiviral particles to knock down mitochondrial transcriptional factor A (TFAM). Null target (NT) and TFAM-knockdown (TFAM-KD) represented the control and knockdown 786-O clones, respectively. Protein or mRNA expression levels of TFAM; mtDNA-encoded NADH dehydrogenase subunit 1 (ND1), ND6 and cytochrome *c* oxidase subunit 2 (COX-2); nuclear DNA (nDNA)-encoded succinate dehydrogenase subunit A (SDHA); v-akt murine thymoma viral oncogene homolog 1 gene (*AKT*)-encoded AKT and v-myc myelocytomatosis viral oncogene homolog gene (*c-MYC*)-encoded MYC; glycolytic enzymes including hexokinase II (HK-II), glucose 6-phosphate isomerase (GPI), phosphofructokinase (PFK), and lactate dehydrogenase subunit A (LDHA); and hypoxia-inducible factors the HIF-1α and HIF-2α, pyruvate dehydrogenase kinase 1 (PDK1), and pyruvate dehydrogenase E1 component α subunit (PDHA1) were analyzed by Western blot or Q-PCR. Bioenergetic parameters of cellular metabolism, basal mitochondrial oxygen consumption rate (mOCR_B_) and basal extracellular acidification rate (ECAR_B_), were measured by a Seahorse XF^e^-24 analyzer. Cell invasiveness was evaluated by a trans-well migration assay and vimentin expression. Doxorubicin was used as a chemotherapeutic agent. The results showed a decrease of mtDNA copy numbers in resected RCC tissues (*p* = 0.043). The TFAM-KD clone expressed lower mtDNA copy number (*p* = 0.034), lower mRNA levels of TFAM (*p* = 0.008), ND1 (*p* = 0.007), and ND6 (*p* = 0.017), and lower protein levels of TFAM and COX-2 than did the NT clone. By contrast, the protein levels of HIF-2α, HK-II, PFK, LDHA, AKT, MYC and vimentin; trans-well migration activity (*p* = 0.007); and drug resistance to doxorubicin (*p* = 0.008) of the TFAM-KD clone were significantly higher than those of the NT clone. Bioenergetically, the TFAM-KD clone expressed lower mOCR_B_ (*p* = 0.009) but higher ECAR_B_ (*p* = 0.037) than did the NT clone. We conclude that a reduction of mtDNA copy number and decrease of respiratory function of mitochondria in RCC might be compensated for by an increase of enzymes and factors that are involved in the upregulation of glycolysis to confer RCC more invasive and a drug-resistant phenotype *in vitro*.

## 1. Introduction

Mitochondria are the organelle responsible for ATP production in human cells [1,2]. Each human cell contains several hundreds to one thousand mitochondria and each mitochondrion harbors 2–10 mitochondrial DNA (mtDNA) copies to form the mitochondrial network [3,4,5,6]. The amount of ATP production is influenced by the number of mtDNA copies and the abundance of mitochondria under different cell types and various physiological conditions [2,6,7].

Human mtDNA (Available online: http://www.mitomap.org/MITOMAP) is a circular structure with 16,569 base-pair (bp) [2,6,7]. It encodes 13 polypeptides that are essential for the assembly of respiratory enzyme Complexes I, III, IV, and V. The other ~90 polypeptides constituting the respiratory enzyme complexes are encoded in nuclear DNA (nDNA). The four subunits of respiratory enzyme Complex II are totally encoded in nDNA. The non-coding region, also called displacement loop (d-loop), is the regulatory region for mtDNA replication and transcription [2,6,7]. Several proteins are involved in the mtDNA replication or transcription, and mitochondrial transcriptional factor A (TFAM) plays dual roles in mtDNA replication and transcription through binding to d-loop. TFAM plays a pivotal role in the regulation of mitochondrial biogenesis [6,8,9].

Human cancers usually exhibit rapid tissue growth beyond the neo-vascularization with subsequent cancer central hypoxia [10,11]. Mitochondrial alterations elicited by hypoxia in tumor microenvironment deserves appraisal. About 80 years ago, Dr. Otto Warburg found that human cancers displayed decreased mitochondrial respiration but increased glycolysis for ATP production during glucose metabolism. He contended that human cancer mitochondria are defective, impaired, or even destroyed. Such a glucose metabolic shift between mitochondrial respiration and glycolysis in human cancers has been coined as Warburg effect [12,13,14].

Alterations of mtDNA copy numbers in human cancers have been extensively studied [15,16,17]. An increase of the mtDNA copy number was noted in head and neck cancers, or esophageal squamous cell carcinoma, especially cigarette smokers [18,19,20,21,22]. Such an increase was supposed to compensate for the damaged mtDNA to keep the supply of ATP by mitochondrial respiration above a threshold. A decrease of mtDNA copy number was noted in hepatic carcinoma, gastric carcinoma, lung cancer, or advanced lung cancer after neoadjuvant chemotherapy [23,24,25,26,27]. Such a decrease of mtDNA copy number would cause the decline of mitochondrial function. Since human kidney harbors abundant blood supply, the newly-grown RCC would suffer from central hypoxia with reduced mtDNA content and impairment of mitochondrial function [10,28,29].

In this study, we analyzed the alterations of mtDNA copy number in resected human RCC kidneys. We used the RCC cell line, the 786-O, to knockdown TFAM expression to decrease mtDNA replication and transcription, and to appraise the alterations of glucose metabolism, aggressiveness, and resistance to anticancer drugs. We have proposed a molecular mechanism to explain the role of mtDNA copy number alterations in the pathophysiology of human RCC.

## 2. Results

### 2.1. Decrease of mtDNA Copy Number in Human RCC Tissues

Compared to the paired non-cancerous counterparts, the mtDNA copy numbers of the five RCC cancerous nests were significantly decreased (0.17 ± 0.06 *vs.* 0.50 ± 0.27, *p* = 0.043, Table 1).

### 2.2. Lower mtDNA Copy Number and Lower Expression Levels of mtDNA-Encoded Polypeptides of Respiratory Enzymes in the TFAM-KD Clone

**Figure 1:**
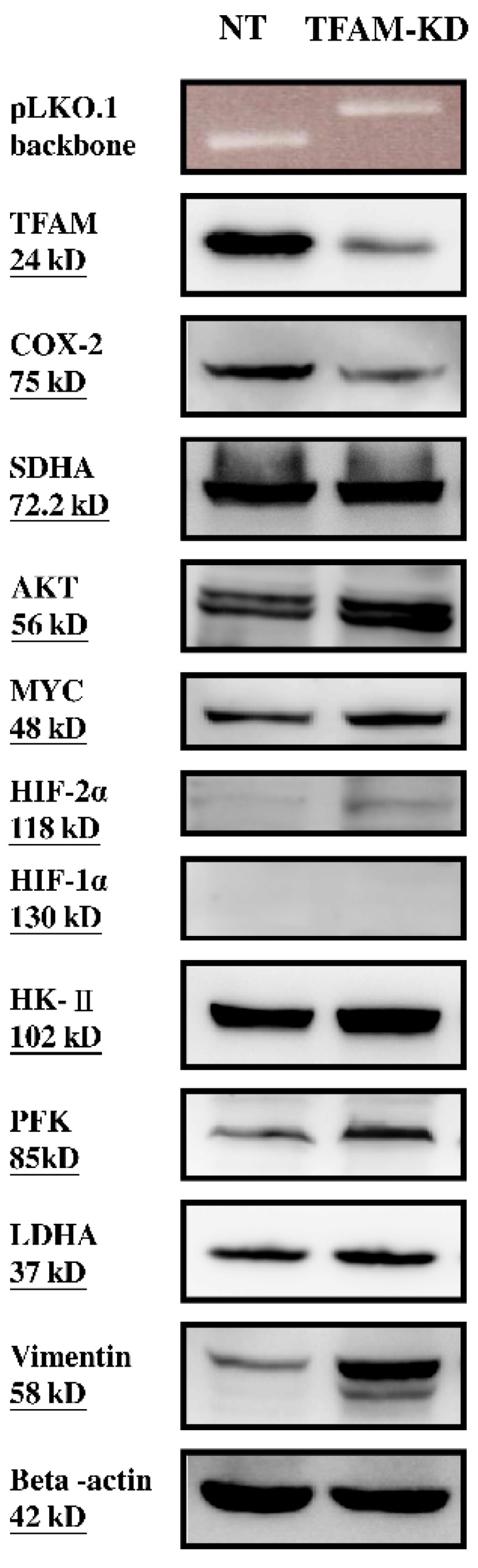
The pLKO.1 backbone was found in the NT and TFAM-KD clones, respectively (the 1st row). Western blot showed that the TFAM-KD clone had lower TFAM (the 2nd row), lower COX-2 (the 3rd row) and similar SDHA (the 4th row); higher AKT (the 5th row), MYC (the 6th row), HIF-2α (the 7th row), HK-II (the 9th row), PFK (the 10th row), LDHA (the 11th row), and vimentin (the 12th row) protein expression levels as compared with those of the NT clone. Both the NT and TFAM-KD clones had no detectable HIF-1α (the 8th row). The expression level of β-actin (the 13th row) was used as an internal control.

The control vector pLKO.1-sh-NT and knockdown vector pLKO.1-sh-TFAM were detected in NT and TFAM-KD clones, respectively (Figure 1). The mRNA expression level of TFAM in the TFAM-KD clone was lower than that of NT clone (0.29 ± 0.15 *vs.* 1.00 ± 0.20, *p* = 0.008, Table 2). Additionally, the protein expression level of TFAM in the TFAM-KD clone was lower than that of the NT clone (Figure 1).

The mtDNA copy number (0.72 ± 0.09 *vs.* 1.00 ± 0.12, *p* = 0.034, Table 2), mRNA expression levels of mtDNA-encoded ND1 (0.55 ± 0.13 *vs.* 1.00 ± 0.07, *p* = 0.007, Table 2), and ND6 (0.51 ± 0.14 *vs.* 1.00 ± 0.16, *p* = 0.017, Table 2), and the protein expression level of mtDNA-encoded COX-2 (Figure 1) of the TFAM-KD clone were lower than those of the NT clone. Nevertheless, there was no obvious difference in the protein expression level of nDNA-encoded SDHA between the TFAM-KD and NT clones (Figure 1).

### 2.3. Higher Expressions of Glycolytic Enzymes in the TFAM-KD Clone

The TFAM-KD clone had higher HK-II mRNA (1.51 ± 0.12 *vs.* 1.00 ± 0.16, *p* = 0.013, Table 2) and protein expression levels (Figure 1), and higher PFK mRNA (1.27 ± 0.16 *vs.* 1.00 ± 0.14, *p* = 0.050, Table 2) and protein expression levels than did the NT clone. Although the LDHA protein expression level in the TFAM-KD clone was higher than that of the NT clone (Figure 1), the difference was not significant in the mRNA expression level (Table 2).

### 2.4. Alterations of Proteins Related to HIF Pathway in the TFAM-KD Clone

Neither TFAM-KD nor NT clone had detectable HIF-1α protein (Figure 1). There were no differences in the mRNA expression levels for PDK1 and PDHA1 between the TFAM-KD and NT clones (Table 2). Interestingly, the protein expression level of HIF-2α in the TFAM-KD clone was higher than that of the NT clone (Figure 1).

### 2.5. Higher Expression of AKT- and cMYC-Encoded Proteins that Enhance Warburg Effect in the TFAM-KD Clone

The protein expression levels of *AKT-* and *c-MYC-*encoded AKT and MYC that enhance Warburg effect [13] in the TFAM-KD clone were higher than those of the NT clone (Figure 1).

### 2.6. Lower OCR and Higher ECAR of Cellular Metabolism in the TFAM-KD Clone

With regard to the oxygen consumption and lactate production during cellular metabolism (Table 3), the TFAM-KD clone had a lower mOCR_B_ (1294.9 ± 187.3 *vs.* 1986.3 ± 167.4 pmole/min/10^6^ cells, *p* = 0.009), a lower maximal mitochondrial oxygen consumption rate (mOCR_Max,_ 1335.1 ± 90.5 *vs.* 2056.8 ± 176.3 pmole/min/10^6^ cells, *p* = 0.003) but a higher ECAR_B_ (2230.2 ± 77.2 *vs.* 2016.4 ± 15.0 mpH/min/10^6^ cells, *p* = 0.037) as compared with those of the NT clone.

To evaluate the alteration of cellular metabolism in the TFAM-KD clone, we measured the mOCR_B_/ECAR_B_ and ECAR_B_/mOCR_B_, respectively, to compare the contribution of aerobic metabolism and anaerobic glycolysis to energy production [30]. Interestingly, the TFAM-KD clone had a lower ratio of mOCR_B_/ECAR_B_ (0.580 ± 0.079 *vs.* 0.983 ± 0.075, *p* = 0.003) but a higher ratio of ECAR_B_/mOCR_B_ (1.747 ± 0.249 *vs.* 1.020 ± 0.078, *p* = 0.009) as compared with those of the NT clone.

### 2.7. Higher Trans-Well Migration Activity and Vimentin Expression in the TFAM-KD Clone

**Figure 2:**
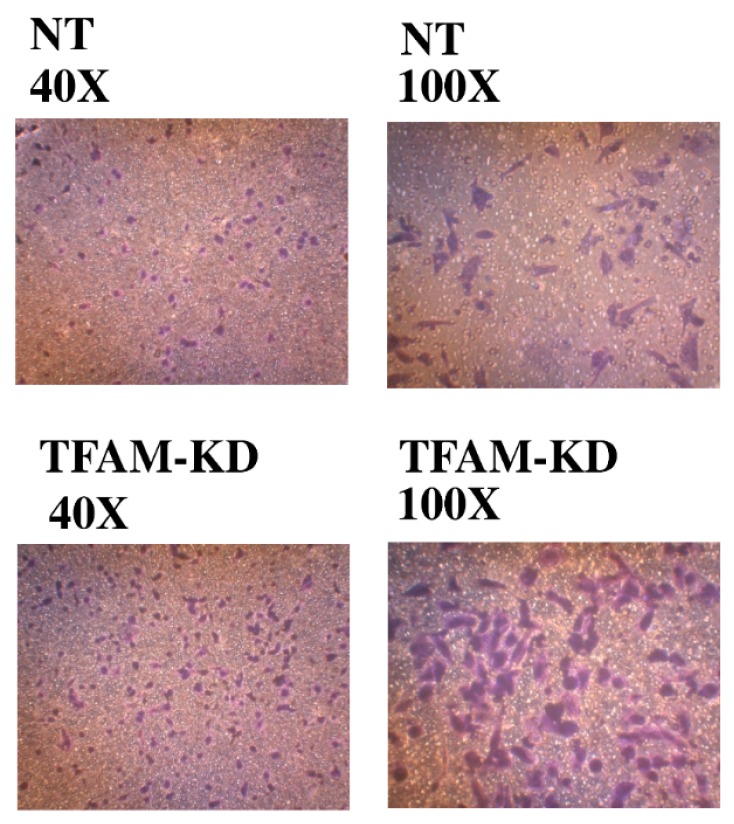
Trans-well migration assay was performed through a piece of membrane with 8-μm pores (Merck Millipore, Billerica, MA, USA). The more cells invading across the pores denotes a higher invasive activity. Under a light microscope (40×, **left side**; and 100×, **right side**), the TFAM-KD clone exhibited a higher trans-well migration activity than did the NT clone.

The TFAM-KD clone had a higher trans-well migration activity (380.3 ± 81.2 *vs.* 132.3 ± 27.5 cells/field, 40×, *p* = 0.007) and a higher protein expression level of vimentin as compared with those of the NT clone (Table 3; Figure 1 and Figure 2).

### 2.8. Higher Drug Resistance to Doxorubicin in the TFAM-KD Clone

The cellular viability of the TFAM-KD clone was higher than that of the NT clone (35.0% ± 2.4% *vs.* 25.1% ± 2.5%, *p* = 0.008) after exposure to doxorubicin (0.5 μM) for 48 h (Table 3). There was no obvious difference in the viability of cells treated with doxorubicin at the concentrations of 1.0 and 2.5 μM, respectively.

## 3. Discussion

During the past 20 years, alterations of mtDNA copy number in several human cancers had been extensively investigated [15,16,17,18,19,20,21,22,23,24,25,26,27,34,35]. Simonnet *et al.* and Meierhofer *et al.* demonstrated a decrease of mtDNA copy number and a decline of mitochondrial enzyme activity in human RCC and such a decrease was associated with the aggressiveness of RCC [29,36]. Similarly, we showed a significant decrease of mtDNA copy number among the five RCC samples in this study (Table 1). Theoretically, the mitochondrial ATP production is positively related to the mtDNA copy number [2,6,7]. RCC tissues with lower mtDNA copy number might display a lower mitochondrial ATP production. However, as proposed by Dr. Warburg, such a decline of mitochondrial respiration could be compensated for by an increased glycolysis [14,37,38]. This scenario has been validated in several types of cancers. However, whether this is the case in RCC awaits further study.

Regarding the regulation of mtDNA copy number, DNA polymerase gamma and TFAM should be emphasized in cancers [6,8]. Previous studies showed some mutations in the gene coding for DNA polymerase gamma or TFAM in breast cancers or colon cancers with low mtDNA copy numbers [39,40]. Since TFAM plays the dual role of mtDNA replication and transcription [6,8,9], we further appraised the role of decreased mtDNA copy number in RCC through the knockdown of TFAM.

Consistent with our expectation, the mtDNA copy number, protein levels of mtDNA-encoded polypeptides (ND1, ND6 and COX-2) and the rates of oxygen consumption (mOCR_B_ and mOCR_Max_) of the TFAM-KD clone were lower than those of the NT clone (Table 2 and Table 3; Figure 1). Undoubtedly, the TFAM-KD clone had significant lower expression levels of proteins involved in mitochondrial biogenesis. Interestingly, the TFAM-KD clone showed higher expression levels of glycolytic enzymes, including HK-II, PFK (the rate-limiting step of glycolysis), and LDHA (Table 2 and Figure 1) [41,42]. This indicates an increase of glycolysis to compensate for the impairment of mitochondrial biogenesis. However, the difference in the LDHA mRNA expression between the NT and TFAM-KD clones was not as obvious as protein expression (Table 2). As a result, additional information is needed to validate the upregulation of glycolysis in the TFAM-KD clone. Since an increase of glycolysis would contribute to the accumulation of lactate, the TFAM-KD clone did exhibit a higher ECAR_B_ value (Table 3). We confirmed that RCC cells had a lower mtDNA copy number, suggesting a decrease of mitochondrial biogenesis, which would be compensated for by an increase of glycolysis. However, the underlying mechanism for the upregulation of glycolysis in RCC warrants further investigation [13,38,43].

Dr. Dang and coworkers have made great efforts to establish the associations among HIF-1α, Warburg effect, and glycolysis in human cancers [10,13,42]. HIF-1α can suppress mitochondrial biogenesis and respiration, and concurrently enhance glycolysis [13,44]. However, in the literature, the 786-O RCC cell line is negative for HIF-1α protein expression [45]. Consistently, TFAM-KD or NT clones derived from 786-O RCC cells were negative for HIF-1α protein expression as revealed in this study (Figure 1). It has been established that HIF-1α, PDK1 and PDH play an important role to regulate mitochondrial biogenesis during hypoxia [13,46]. Without the signaling from the upstream HIF-1α, it is reasonable that we observed no obvious difference in the mRNA expression levels of PDK1 and PDHA1 between the NT and TFAM-KD 786-O clones. Interestingly, the TFAM-KD clone had a higher protein expression level of HIF-2α (Figure 1). Although HIF-1α and HIF-2α have some overlapping effects, they regulate distinct cellular functions [47]. HIF-1α primarily participates in the upregulation of glycolysis [48,49]. HIF-2α is mainly involved in the regulation of tumor growth and cell cycle progression (Figure 1 and Figure 2) [48,50]. As a result, the higher expression of HIF-2α in the TFAM-KD clone might confer its higher invasive activity (Table 3, trans-well migration activity). However, the signaling pathways that lead to the upregulation of glycolysis in the TFAM-KD clone has remained unknown.

Several oncogenes have been implicated in the upregulation of glycolysis of human cancers, including *AKT* and *c-MYC* [13]. AKT can mobilize glucose transporters to the cell surface and activate HK-II to enhance glycolysis [51]. MYC can activate most of the genes coding for glycolytic enzymes and directly binds to numerous glycolytic genes, including those encoding HK-II, enolase, and LDHA [42]. Both AKT and MYC may enhance the Warburg effect in human cancers. Interestingly, we observed that the TFAM-KD clone not only expressed higher levels of AKT and MYC, but also showed higher expression levels of glycolytic enzymes including HK-II, PFK, and LDHA (Figure 1). These findings led us to conclude that AKT and MYC, in addition to HIF-1α, play important roles to upregulate glycolysis in RCC 786-O cells.

Recently, the role of mechanistic target of rapamycin (mTOR) signaling through mTOR complex 1 (mTORC1) or mTORC2 in regulating human protein translation and ribosome biogenesis have been extensively investigated [52], and their roles in the TFAM-KD 786-O RCC cells with mitochondrial dysfunction deserved discussion. Xu *et al.* reported that stimulation of mTORC1 with l-leucine increased the efficiency of mitochondrial transcription and translation to improve the mitochondrial function in Robert syndrome, a human developmental disorder [53,54]. Morita *et al.* further demonstrated that mTORC1 could stimulate the function and biogenesis of mitochondria through the upregulation of TFAM translation [55]. Additionally, Morita *et al.* and Masui *et al.* also showed that mTORC2 could stimulate glycolysis though activation of AKT and MYC [55,56]. In this study, we found that the expression levels of AKT and MYC were increased in the TFAM-KD 786-O RCC cells. We thus speculate that the mTOR signaling could orchestrate the metabolic shift in the TFAM-knockdown 786-O cells examined in this study [57]. However, further studies are warranted to establish the signaling cascade.

The role of glycolysis in the resistance to anticancer drug, which is an important characteristic of malignancies, has been investigated [58,59]. Since an increase of glycolysis not only provides ATP production, the off-shoot pentose phosphate pathway also offers ribose and NADPH for the biosynthesis of nucleotides and proteins, cell proliferation and protection from oxidative damage [60]. It is reasonable to explain that the increased glycolysis might render the TFAM-KD clone higher proliferation rate and higher resistance to oxidative damages caused by doxorubicin [61].

Cancer stem cells have a highly invasive activity and are highly resistant to chemotherapeutic agents, and the surface markers identification are the standard methods to select cancer stem cells [62]. Recently, the association between the cancer cell stemness and Warburg effect has been discussed and that the glucose metabolic reprogramming is considered an important biological hallmark of cancer stemness [63,64]. Chen *et al.* reported that hypoxia might confer cancer cells to gain stemness and Shen *et al.* further demonstrated that a decrease of OCR/ECAR ratio, indicating that a metabolic shift, might render cancer cells the specific feature of stemness [30,65]. Furthermore, Guha *et al.* reported that mtDNA reduction may drive the generation of breast cancer stem cells [66]. It is of interest to note the effect of TFAM knockdown on the stemness of cancer cells. Interestingly, the TFAM-KD clone expressed a decrease in mOCR_B_ (indicating conditions that mimic hypoxia) and mOCR_B_/ECAR_B_ ratio (indicating metabolic reprogramming), and displayed a higher trans-well migration activity, higher resistance to doxorubicin, and a higher level of vimentin expression. These findings and other preliminary results suggest that TFAM knockdown could induce the stemness of human renal cancer cells. However, we did not evaluate the surface markers of the TFAM-KD clone, further studies have been designed to validate these observations.

## 4. Materials and Methods

### 4.1. Collection of Clinical Samples and DNA Extraction

Surgical resected kidneys from 5 RCC patients were collected from the Division of Urology, Taipei Hospital, Ministry of Health and Welfare, New Taipei City, Taiwan. Their pathological slides were reviewed carefully. Representative tumor foci without tumor necrosis and without lymphocyte infiltration were identified, and thin slices about 5 μm in thickness from paraffin-embedded tissue blocks were prepared for DNA extraction. Simultaneously, paired non-cancerous renal tissues were also collected. After the de-wax and re-hydration processes, the tissue samples were mixed with 200 μL of DNA extraction solution (QuickExtract, Epicenter, Madison, WI, USA) to extract total cellular DNA at 65 °C for 3 h as described previously [20,21,35]. The DNA sample was kept at −20 °C until use. This study was approved by the Institution Review Board of Taipei Hospital, Ministry of Health and Welfare (IRB No.: TH-IRB-0016-0005).

### 4.2. RCC Cell Line

We purchased the 786-O RCC cell line from the Food Industry Research and Development Institute in Taiwan (Available online: http://www.firdi.org.tw/) to perform the experiments reported herein. The culture medium was composed of Dulbecco’s modified Eagle’s medium (DMEM) plus 10% of fetal bovine serum (FBS) and 1% of a mixture of Penicillin G and streptomycin sulfate [45].

### 4.3. Viral Infection to Knockdown TFAM Expression

A small hairpin RNA (sh-RNA) designed from the National RNAi Core Facility of Academia Sinica, Taiwan (Available online: http://rnai.genmed.sinica.edu.tw/index) was applied to knockdown the expression of TFAM. A vector derived from the pLKO.1 backbone harboring a specific sh-oligonucleotide with the sequence against TFAM mRNA was packaged into lentiviral particles to infect the 786-O RCC cells. The target sequence against TFAM gene is 5′-CGTTTATGTAGCTGAAAGATT-3′. For comparison, a null target (NT) sequence of 5′-TCAGTTAACCACTTTTT-3′ was used as the control [34]. After 5 MOI (multiplicity of infection) of viral infection, the 786-O RCC cell harboring the pLKO.1-sh-NT vector was named as NT clone (control clone) and the RCC 786-O cell harboring the pLKO.1-sh-TFAM vector was named as TFAM-KD clone (knockdown clone). Comparative analyses of the mRNA and protein expression levels of genes of interest, trans-well migration, and anticancer drug resistance were performed between the NT and TFAM-KD clones.

### 4.4. DNA, RNA, and Protein Extractions

DNA extraction. Genomic DNA was purified by standard procedures using Tris-EDTA buffer, sodium dodecyl sulfate and proteinase K followed by phenol/chloroform extraction [34,67,68]. The DNA pellet was dissolved in distilled water and kept at −20 °C until use.

RNA extraction. For total RNA extraction, cells were lysed with TRI^™^ Reagent (Sigma-Aldrich Chemical Co., St. Louis, MO, USA) according to the manufacturer’s instructions. Then, 2 μg of purified RNA was reversed-transcribed to cDNA with the Ready-to-Go RT-PCR kit (GE Healthcare, Chicago, IL, USA) by using appropriate oligo-dT primers [34,67,68].

Protein extraction. Cell lysates were prepared using a lysis buffer (50 mM Tris-HCl, 0.25% sodium deoxycholate, 150 mM NaCl, 1 mM EDTA, 1% Triton X-100, and 1% NP-40, pH 7.4) containing 1% of protease inhibitor (Roche Applied Sciences, Penzberg, Germany) and incubated at 4 °C for 30 min, and then centrifuged at 12,000× *g* for 20 min at 4 °C. Total cellular proteins were collected and kept at −80 °C until use [34].

### 4.5. Confirmation of pLKO.1-NT Vector in NT Clone and pLKO.1-sh-TFAM Vector in the TFAM-KD Clone

PCR was executed to confirm the pLKO.1-sh-NT vector in NT clone and the pLKO.1-sh-TFAM vector in the TFAM-KD clone, respectively. Each PCR reaction mixture (50 μL) contained 25 μL of RBC SensiZyme^®^ Hot start *Taq* Premix (RBC Bioscience, New Taipei City, Taiwan), 19.5 μL of PCR-grade H_2_O, 2.5 μL of DMSO, 1 μL of each primer (pLKO.1, Table S1), and 1 μL of TFAM-KD or NT DNA (100 ng/μL) to undergo a hot start at 95 °C for 10 min, 40 cycles of 95 °C for 15 s, 58 °C for 15 s, and 72 °C for 30 s, and a final extension at 72 °C for 7 min. PCR products were subject to electrophoresis on a 3% agarose gel to separate the DNA bands (the pLKO.1-NT vector is slightly smaller than the pLKO.1-TFAM vector), which were visualized by ultraviolet light illumination after ethidium bromide staining.

### 4.6. Analysis of mtDNA Copy Number, mRNA and Protein Expression Levels

Q-PCR using SYBR Green I (Roche Applied Science, Penzberg, Germany) was carried out to determine the threshold cycle (*C*_t_) to determine the mtDNA copy number and mRNA expression levels of specific target genes [34,67,68]. The mtDNA copy number is defined as the number of total tRNA^Leu^ copies of mtDNA divided by the number of total 18S rRNA copies of nDNA after adjusting with the mtDNA copy number of the 143B cells as 1.00 [20,21,26,34]. The mRNA expression level was defined as the number of total target gene cDNA copies divided by the number of total 18S rRNA cDNA copies after adjusting with mRNA expression level of the target gene in the 143B cells as 1.00 [67,68]. Each experiment was done in duplicate to get the average value, and was repeated for three generations of sub-cultures in cell line study (*n* = 3). Concerning the mtDNA copy number and mRNA expression levels in the cell line study, the mean (M) of the NT clone was adjusted to 1.00 in data presentation [68]. The replication efficiencies of primers were established by using the 143B cells as previously reported [68], and the sequences of primers are summarized in supplementary materials Table S1.

The relative protein expression levels were determined by Western blot [34]. An aliquot of 50 μg of total cellular protein was separated on a 10% SDS-PAGE, and then blotted onto a piece of BioTrace™ polyvinylidenedifluoride (PVDF) membrane (Pall Corp., Pensacola, FL, USA). Non-specific bindings were blocked with 5% skim milk in Tris-buffered saline Tween-20 (TBST) buffer (50 mM Tris-HCl, 150 mM NaCl, 0.1% Tween-20, pH 7.4). The membrane was subjected to specific primary antibodies against (1) mitochondrial biogenesis-related proteins, including TFAM (Cell Signaling, Danvers, MA, USA; 1:1000, 24 kD), COX-2, a subunit of mtDNA-encoded respiratory enzyme Complex IV (GeneTex, Irvine, CA, USA; 1:500, 75 kD) and SDHA, a subunit of nDNA-encoded respiratory enzyme Complex II (Molecular Probes, Eugene, OR, USA; 1:1000, 72.2 kD); (2) HIF pathway-related proteins, including HIF-1α (GeneTex, 1:1000, 130 kD) and HIF-2α (GeneTex, 1:1000, 118 kD); (3) oncogene, *AKT*, and *c-Myc*, encoded proteins that enhance the Warburg effect [13,69,70], including AKT (GeneTex, 1:1000, 56 kD) and MYC (GeneTex, 1:1000, 48 kD); (4) glycolytic enzymes, including HK-II (Merck Millipore, 1:5000, 102 kD), PFK (GeneTex, 1:500, 85 kD), and LDHA (Cell Signaling, 1:1000, 37 kD); (5) mesenchymal marker for epithelial mesenchymal transition (EMT), the vimentin (Sigma, 1:1000, 58 kD). Beta-actin (Merck Millipore; 1:10,000, 42 kD) was used as the control. The membrane was incubated with an electrochemical luminescence reagent (Omics Biotechnology Co., Taipei, Taiwan) and the intensity of the target protein band was visualized by using Image Quant™ LAS4000 (GE Healthcare Life-Sciences Ltd., Tokyo, Japan).

### 4.7. Analysis of Bioenergetic Parameters by the XF^e^-24 Analyzer

The bioenergetic parameters, the oxygen consumption rate (OCR) and extracellular acidification rate (ECAR), were determined on a Seahorse XF^e^-24 Analyzer (Seahorse Bioscience, Billerica, MA, USA) according to the manufacturer’s instructions [71]. During steady stage, the basal OCR (OCR_B_), and basal ECAR (ECAR_B_) were determined. Then, oligomycin (OM, Complex V inhibitor, 1 µM), carbonyl cyanide-*4*-(trifluoromethoxy)-phenylhydrazone (FCCP, an uncoupling agent of mitochondrial respiration to achieve the maximal respiration rate, 0.3 µM), and rotenone/antimycin A (RA, Complex I/III inhibitor, 0.5 µM) were added to get the OCR_OM_, OCR_FCCP_ and OCR_RA_ in order. The OCR_RA_ denotes the non-mitochondrial OCR. The mOCR_B_ was defined as OCR_B_-OCR_RA_ and the mOCR_Max_ was defined as OCR_FCCP_-OCR_RA_ [71]. The OCR_B_, OCAR_OM_, OCR_FCCP_, and OCR_RA_ were recorded at three time points within a time period to get their average, and experiments were performed using three independent generations of subcultures (*n* = 3). All the data are presented as M ± S.D.

### 4.8. Trans-Well Migration Activity Assay

Trans-well cell migration activity was assayed by using a 24-well culture plate and a millicell hanging cell culture insert, which was covered with a piece of membrane with 8-μm pores (Merck Millipore) as described previously [34]. The more cells invading across the pores denoted a higher invasive activity. Three random areas under a light microscope (40×) were selected to count the invaded cells to get the average number (cells/field) [34]. Each experiment was performed using three independent subcultures (*n* = 3). The data are presented as M ± S.D.

### 4.9. Drug Resistance to Doxorubicin

A total of 5000 cells suspended in 100 μL of growth medium were seeded on a 96-well microplasty reader plate (Corning Glass Works, Corning, NY, USA) for 24 h. The growth medium was then changed to new ones with and without the addition of doxorubicin (0.50, 1.0 and 2.5 μM, respectively), as executed for 786-O RCC cell line in the literatures. The concentration of 0.50 μM was the optimal dose, because at 1.0 μM and 2.5 μM doxorubicin was lethal to the cells [31,32,33]. After incubation for 48 h at 37 °C, an additional 200 μL of 1× AlamarBlue™ reagent (Invitrogen, Waltham, MA, USA) was added to the cells and incubated for 1.5 h. The fluorescence intensity was measured by the Victor^2^™1420 Multilabel Counter (Perkin-Elmer, Waltham, MA, USA) on a plate reader at the excitation wavelength of 538 nm and emission wavelength of 590 nm [34]. The cell viability was calculated by the ratio between the fluorescence intensity of cells treated with doxorubicin and that of cells without treatment with doxorubicin. Each experiment was done in duplicate and repeated for three independent subcultures (*n* = 3). The data are presented as M ± S.D.

### 4.10. Statistical Analysis

The continuous variables between two matched groups or two independent groups were compared by using paired *t*-test, Wilcoxon signed ranks test, Student’s *t-*test, or Mann-Whitey *U* test, when appropriate. Significance was considered when a *p* value was less than 0.05.

## 5. Conclusions

In conclusion, a decrease of mtDNA copy number was observed in human RCC tissues. Such a decrease in mtDNA copy number and mitochondrial dysfunction might be compensated for by an increase of glycolysis, *i.e.*, the Warburg effect, which confers RCC with greater invasiveness and a phenotype of higher drug resistance. Further studies are warranted to investigate whether such a decrease of mtDNA copy number enables RCC to have properties of cancer stem cells.

## Figures and Tables

**Table 1:** Comparison of mtDNA copy number between the non-cancerous and cancerous parts of the resected kidneys from five patients with renal cell carcinoma (RCC).

	mtDNA Copy Number *		*p*-Value **
	Non-Cancerous Part	Cancerous Part	
Overall (*n* = 5)			
M ± S.D.	0.50 ± 0.27	0.17 ± 0.06	0.043
Examined subjects			
Patient 1	0.22	0.12	-
Patient 2	0.25	0.14	-
Patient 3	0.81	0.15	-
Patient 4	0.49	0.28	-
Patient 5	0.74	0.16	-

* The mtDNA copy number of the 143B cells was defined as 1.00; ** Paired *t*-test or Wilcoxon signed ranks test was used when appropriate; M, mean; S.D., standard deviation.

**Table 2:** The differences in mtDNA copy number and mRNA expression levels between the 786-O RCC NT and TFAM-KD clones.

Parameters	786-O RCC (*n* = 3)		*p*-Value **
	NT	TFAM-KD	
mtDNA copy number (M ± S.D.) *	1.00 ± 0.12	0.72 ± 0.09	0.034
mRNA expression level (M ± S.D.) *			
TFAM	1.00 ± 0.20	0.29 ± 0.15	0.008
ND1	1.00 ± 0.07	0.55 ± 0.13	0.007
ND6	1.00 ± 0.16	0.51 ± 0.14	0.017
PDK1	1.00 ± 0.41	1.08 ± 0.43	0.830
PDHA1	1.00 ± 0.38	0.98 ± 0.40	0.947
HK-II	1.00 ± 0.16	1.51 ± 0.12	0.013
GPI	1.00 ± 0.10	0.93 ± 0.25	0.664
PFK	1.00 ± 0.14	1.27 ± 0.16	0.050
LDHA	1.00 ± 0.33	1.32 ± 0.70	0.268

* The mtDNA copy number and target gene mRNA expression of the 143B cells was defined as 1.00 during calculation, and then the mean (M) of the NT clone was adjusted to 1.00 in data presentation; ** *t*-test or Mann-Whitey *U* test was used when appropriate; abbreviations: M, mean; S.D., standard deviation; TFAM, mitochondrial transcriptional factor A; ND1, NADH dehydrogenase subunit 1; ND6, NADH dehydrogenase subunit 6; PDK1, pyruvate dehydrogenase kinase 1; PDHA1, pyruvate dehydrogenase E1 component α subunit; HK-II, hexokinase II; GPI, glucose 6-phosphate isomerase; PFK, phosphofructokinase; LDHA, lactate dehydrogenase subunit A.

**Table 3:** The differences in OCR and ECAR of cellular metabolism, trans-well migration activity and drug resistance to doxorubicin between the 786-O RCC NT and TFAM-KD clones.

Parameters	786-O RCC (*n* = 3)		*p*-Value *
	NT (M ± S.D.)	TFAM-KD (M ± S.D.)	
OCR of cellular metabolism			
mOCR_B_ (pmole/min/10^6^ cells)	1986.3 ± 167.4	1294.9 ± 187.3	0.009
mOCR_Max_ (pmole/min/10^6^ cells)	2056.8 ± 176.3	1335.1 ± 90.5	0.003
ECAR of cellular metabolism			
ECAR_B_ (mpH/min/10^6^ cells)	2016.4 ± 15.0	2230.2 ± 77.2	0.037
Cellular metabolic shift			
mOCR_B_/ECAR_B_	0.983 ± 0.075	0.580 ± 0.079	0.003
ECAR_B_/mOCR_B_	1.020 ± 0.078	1.747 ± 0.249	0.009
Trans-well migration activity (cells/field)	132.3 ± 27.5	380.3 ± 81.2	0.007
Relative cell viability (%) **			
Doxorubicin concentration [31,32,33]			
0.5 μM	25.1 ± 2.5	35.0 ± 2.4	0.008
1.0 μM	11.5 ± 3.7	12.8 ± 3.0	0.513
2.5 μM	10.9 ± 4.8	14.5 ± 2.5	0.275

* *t*-test or Mann-Whitey *U* test was used when appropriate; ** number of survived cells with doxorubicin treatment at 48 h/number of survived cells without doxorubicin treatment at 48 h; M, mean; S.D., standard deviation; OCR, oxygen consumption rate; mOCR_B_, basal mitochondrial OCR; mOCR_Max_, maximal mitochondrial OCR; ECAR, extracellular acidification rate; ECAR_B_, basal ECAR.

## Supplementary Materials

Supplementary materials can be found at http://www.mdpi.com/1422-0067/17/6/814/s1.
