# Supplementary material for: Role of Mitochondrial DNA Copy Number Alteration in Human Renal Cell Carcinoma
Source: Int J Mol Sci. 2016 May 25;17(6):814. doi: 10.3390/ijms17060814 (PMC4926348; doi:10.3390/ijms17060814)
Supplement: Supplementary file 1 [file ijms-17-00814-s001.pdf]

Supplementary Materials: Role of Mitochondrial DNA Copy Number Alteration in Human Renal Cell Carcinoma

Chen-Sung Lin, Hui-Ting Lee, Ming-Huei Lee, Siao-Cian Pan, Chen-Yeh Ke, Allen Wen-Hsiang Chiu and Yau-Huei Wei

Table S1. Summary of the analyzed target genes and oligonucleotide sequences of the primers used to amplify the indicated genes.

DNA	Forward Primer Sequence	Reverse Primer Sequence
mtDNA [1]	CACCCAAGAACAGGGTTTGT	TGGCCATGGGTATGTTGTTAA
nDNA [2]	TAGAGGGACAAGTGGCGTTC	CGCTGAGCCAGTCAGTGT
pLKO.1	ACAAAATACGTGACGTAG	CTGTTGCTATTATGTCTAC
**Messenger RNA (mRNA)**	**Forward Primer Sequence**	**Reverse Primer Sequence**
**Reference Gene**		
18S rRNA	CTCAACACGGGAAACCTCAC	CGCTCCACCAACTAAGAACG
**Target Genes**		
TFAM	CAACTACCCATATTTAAAGCTCAGAA	GAATCAGGAAGTTCCCTCCA
ND1	CCACCCTTATCACAACACAAGA	TCATATTATGGCCAAGGGTCA
ND6	GGTGCTGTGGGTGAAAGAGT	AACCCTGACCCCTCTCCTT
PDK-1	CAAGACCTCGTGTTGAGACCT	ACGTGATATGGGCAATCCAT
PDHA1	TCCGAGAGGCAACAAGGTT	AAGTCTGCAGCTCCATCAGG
HK-II	CCCTGCCACCAGACTAAACT	TGGACTTGAATCCCTTGGTC
GPI	GGAAGGGTCTGCATCAAAG	CCTCATCAGGGCCTCTGTC
PFK	AGGAGGGGAAGGGCATCT	TTCCTATCAAATGGGGTTGG
LDHA	GCAGATTTGGCAGAGAGTATAATG	GACATCATCCTTTATTCCGTAAAGAC

[1] The mtDNA analyzed region was $tRNA^{Leu}$ gene; [2] The nDNA analyzed region was 18S rRNA gene; TFAM, mitochondrial transcriptional factor A; ND1, NADH dehydrogenase subunit 1; ND6, NADH dehydrogenase subunit 6; PDK1, pyruvate dehydrogenase kinase 1; PDHA1, pyruvate dehydrogenase E1 component alpha subunit; HK-II, hexokinase II; GPI, glucose 6-phosphate isomerase; PFK, phosphofructokinase and LDHA, lactate dehydrogenase subunit A.
